# Associations between item characteristics and statistical performance for paediatric medical student multiple choice assessments

**DOI:** 10.12688/mep.19764.1

**Published:** 2023-11-08

**Authors:** Isabelle Bosi, Deborah O'Mara, Tyler Clark, Nounu Sarukkali Patabendige, Sean E. Kennedy, Hasantha Gunasekera

**Affiliations:** 1 Department of Clinical Immunology and Allergy, Royal Prince Alfred Hospital, Camperdown, NSW, 2050, Australia; 2 Children's Hospital at Westmead Clinical School, The University of Sydney, Westmead, New South Wales, 2145, Australia; 3 Education Office, Sydney Medical School, The University of Sydney, Sydney, New South Wales, 2006, Australia; 4 School of Clinical Medicine, Discipline of Paediatrics & Child Health, University of New South Wales, Sydney, New South Wales, 2052, Australia

**Keywords:** Educational Measurement, Multiple choice question, Pediatrics, Program Evaluation, Medical Students, Assessment.

## Abstract

**Background:** Multiple choice questions (MCQs) are commonly used in medical student assessments but often prepared by clinicians without formal education qualifications. This study aimed to inform the question writing process by investigating the association between MCQ characteristics and commonly used statistical measures of individual item quality for a paediatric medical term.

**Methods:** Item characteristics and statistics for five consecutive annual barrier paediatric medical student assessments (each n=60 items) were examined retrospectively. Items were characterised according to format (single best answer vs. extended matching); stem and option length; vignette presence and whether required to answer the question, inclusion of images/tables; clinical skill assessed; paediatric speciality; clinical relevance/applicability; Bloom’s taxonomy domain and item flaws. For each item, we recorded the facility (proportion of students answering correctly) and point biserial (discrimination).

**Results:** Item characteristics significantly positively correlated (p<0.05) with facility were relevant vignette, diagnosis or application items, longer stem length and higher clinical relevance. Recall items (e.g., epidemiology items) were associated with lower facility. Characteristics significantly correlated with higher discrimination were extended matching question (EMQ) format, longer options, diagnostic and subspeciality items. Variation in item characteristics did not predict variation in the facility or point biserial (less than 10% variation explained).

**Conclusions:** Our research supports the use of longer items, relevant vignettes, clinically-relevant content, EMQs and diagnostic items for optimising paediatric MCQ assessment quality. Variation in item characteristics explains a small amount of the observed variation in statistical measures of MCQ quality, highlighting the importance of clinical expertise in writing high quality assessments.

## Introduction

Multiple choice questions (MCQs) are a common assessment modality in health professional student and vocational training. MCQs have multiple advantages including ease of preparation, adaptability to a range of content, familiarity and amenability to automated marking ^1^. MCQs can evaluate student knowledge, including higher-order thinking ^2^. However, poorly written items may favour the ‘exam-wise’ candidate rather than fulfil the goals of assessment.

The 2018 Consensus framework for good assessment ^3^ articulates the features of an optimal single assessment including validity, reproducibility, result equivalence across sites or testing periods, feasibility, educational effect, catalytic effect and stakeholder acceptability. Favourable MCQ attributes include topic salience, option homogeneity and the absence of construction flaws (e.g., negative questions, absolute terms and logical or grammatical cueing) ^4, 5^. Much has been written about item optimisation ^4– 7^, however, existing MCQ construction guidelines are largely derived from consensus rather than quantitative evidence. The process of preparing and reviewing MCQs is labour intensive and often depends on authors with expertise in content rather than medical education, resulting in wide variability in item statistical performance ^8^. Psychometric analysis can help to identify flawed items. Commonly used indices ^9^ include item facility (proportion answering correctly) and discrimination (e.g., ‘point biserial’ measure of correlation between item score with total test score).

Some studies explore the effect of individual item characteristics, e.g., vignettes ^10^ and images ^11^ on item performance. However, few examine the influence of multiple item characteristics to provide evidence-based recommendations for clinicians preparing MCQs for medical students ^12^.

This retrospective study addressed three research objectives using a convenience sample of five 60 item MCQ annual summative paediatric assessments undertaken by medical students in the final or penultimate year of an Australian graduate-entry medical program:

1. Identify how item statistical performance (facility and point biserial) varies according to item characteristics.
2. Determine the item characteristics that are most predictive of statistical performance.
3. Evaluate statistical performance indicators across testing periods.

## Methods

### Ethics statement

This study is governed by the umbrella ethics of the Education Office of the University of Sydney (HREC 2016/456).

### Study context

This retrospective study investigated the characteristics and statistical performance of all 300 MCQs from five high-stakes end-of-year annual summative examinations (2015 to 2019 inclusive), all pre coronavirus disease 2019 (COVID-19) pandemic. Each examination comprised 60 MCQs with the item statistics being based on results from almost 300 students each year; 2015 n=282; 2016 n=274; 2017 n=297; 2018 n=288 and 2019 n=299. The MCQs were either single best answer (SBA) with 5 options, or extended matching questions (EMQ) with up to 15 options. As some items were re-used across the years as anchor questions, unique question ‘items’ and ‘usages’ are reported separately.

As part of our institution’s quality assurance procedures, we review and edit items prior to use and post-administration, then all item statistics are recorded per occasion in our in-house database. Criteria for post administration review include facility <20% and negative point biserial. There were 5 items (7 usages) excluded post-examination, primarily due to item difficulty. As the final item performance statistics are calculated after item exclusion, this study focuses on items retained in the assessments. Therefore, our dataset analysed 173 unique items with 293 usages. For Research Objective 1, we analysed both the 173 unique items and 293 usages to ensure that usage frequency did not affect results. For Research Objectives 2 and 3, the unit of analysis was 293 usages.

### Content analysis of item characteristics

Authors 1, 4, 6 reviewed the medical education literature ^4– 6, 9– 12^ to determine potentially relevant item characteristics to analyse. Characteristics examined in this study were: stem word count (including tabulated words and numbers); number of options; option length (total word count of all options); item type (SBA or EMQ); image or table used (yes/no); clinical vignette present (yes/no); clinical vignette required to answer (yes/no); clinical skill assessed (epidemiology, history, examination, investigation, diagnosis, management, prognosis, research, not applicable); Blooms taxonomy domain (recall, understanding, application); paediatric speciality (subspeciality, community, surgery, public health); clinical relevance (low, moderate, high); total number and types of item writing flaws. This study designed a three-option coding of clinical relevance: low (e.g., basic sciences, research theory), moderate (e.g., epidemiology, potentially esoteric but clinically relevant) and high (e.g., assessing ability to diagnose, investigate or manage common problems). The application of Bloom’s taxonomy was similar to previous descriptions ^5, 11^.

Two junior (paediatric trainees) and one senior academic clinician (Block Coordinator) independently coded the items. Disagreements were managed as follows: all counted (item flaws); majority decision (Bloom’s taxonomy, presence and utility of vignette); consensus (clinical skill assessed) and median score (clinical relevance on a 3-point scale). A further senior clinician from a different academic institution (author 5) coded 20% of the sample (all 2019 items) as a bias check.

Some variables were recoded for analysis as their frequency was too low to warrant individual statistical analysis (i.e., fewer than 10% of items). Recoded variables included: clinical vignette present and required (i.e., question cannot be answered without vignette), clinical skill assessed (basic clinical skill, diagnosis, management, non-clinical) and paediatric subspeciality (general, subspeciality, surgery). Only one item had two item flaws so this became a binary variable (flaws present yes/no). There were insufficient images and tables to warrant analysis of these item characteristics.

Some of the item characteristics were highly intercorrelated (r≥0.75), thereby measuring the same item attribute. ‘Number of options’ and ‘non-clinical skill’ were excluded due to positive correlation with item type (i.e., SBA) and an inverse correlation with ‘high’ clinical relevance respectively. Outliers were identified for stem and option length using a threshold of 1.5 times above/below the interquartile range and recoded to the upper/lower fences ^13– 15^.

### Item statistical performance indicators

Item facility and point biserial results were obtained from the University’s assessment database. Consideration was given to using the Rasch measure of item difficulty, but these were highly intercorrelated with facility (>0.99) and less easily interpretable. As the reliability of point biserials are questioned at the extremes of facility (i.e., <20% or >80%), for the purpose of analyses, we also classified items as ‘low’ or ‘moderate to high’ difficulty based on facility >80% or ≤80% respectively, comparable to other publications ^9, 12^. Most (64% of items and 68% of item usages) were of moderate to high difficulty. We also classified items into two groups based on the point biserial being ≥0.15, which accounts for 31% of items and 37% of item usages. Other authors ^12^ have used a higher figure for their classification but for our homogeneous medical school cohort, the point biserial typically averages 0.10.

### Statistical methods

Research Objective 1 was evaluated through linear correlations with original values and Chi-Square analyses using the binary classification of facility and point biserial outlined above. ANOVA was used to test the differences between means. Research Objectives 2 and 3 were assessed using regression and descriptive analyses respectively. All analyses were conducted using *SPSS Version 28 (IBM Corp. Released 2021)*.

## Results

### Reviewer agreement of item characteristics and flaws

**Figure 1.**
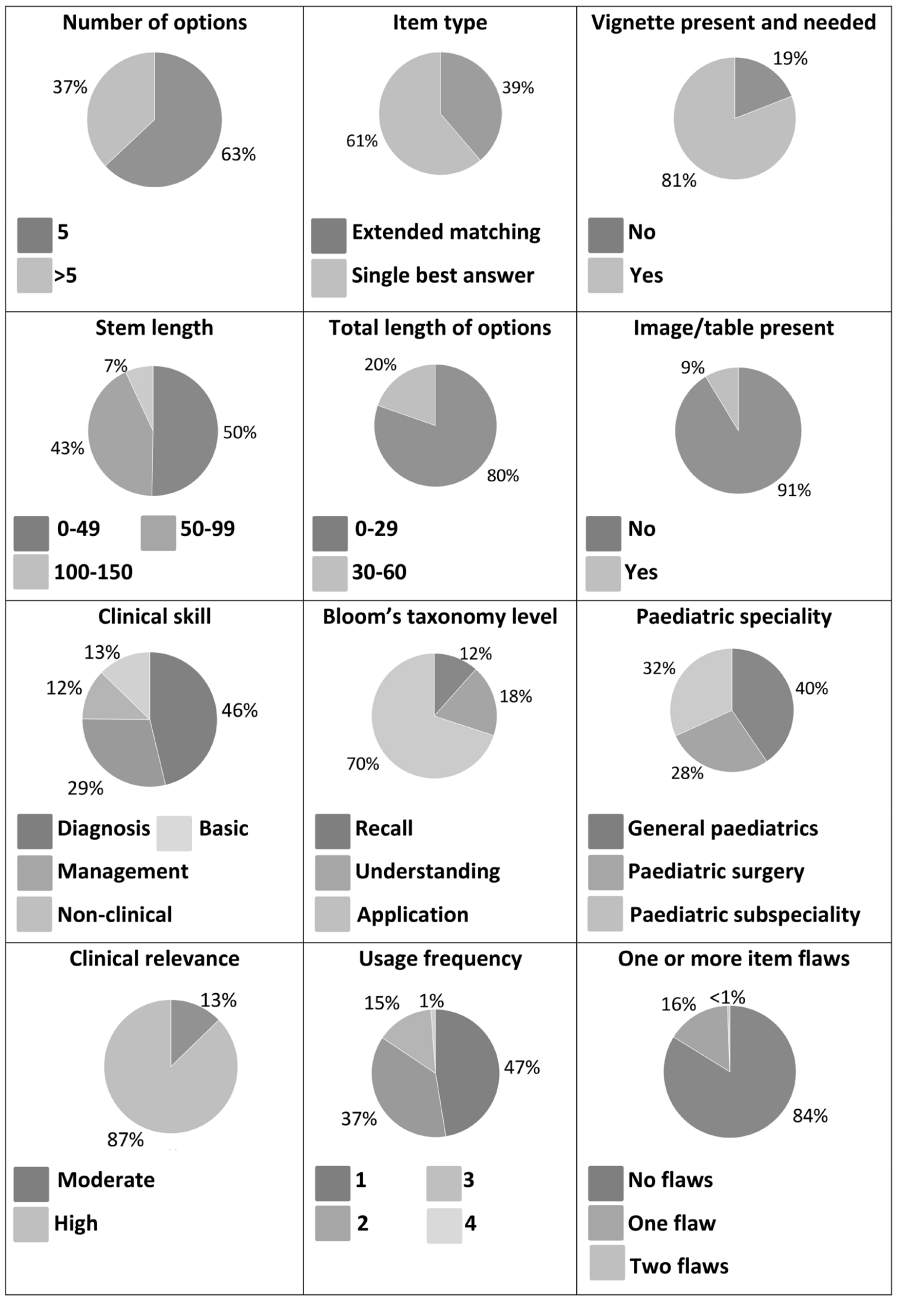
Characteristics of the 173 unique items analysed. Five consecutive annual barrier paediatric medical student assessments (each n=60 items) were examined retrospectively and their construction characteristics were coded by 3 item reviewers. The distribution of item characteristics is summarised for the 173 unique items coded.

There was unanimous agreement between all 3 coders for 85% of the initial coding (67% of the subjective characteristics). 40% of the disagreements represented a one level difference in Bloom’s taxonomy or clinical relevance. Including the independent coder resulted in 83% initial agreement across 4 coders (62% for subjective characteristics). The number of item flaws identified were: none (84% of items); one (16%); or two (<1%). Almost all item flaws (93%) were identified by the same coder (one of the two trainees). Figure 1 summarises item characteristics.

### Item statistical performance measures

All underlying data can be found on Figshare ^16– 18^.

The item facility ranged from 14 – 100% (By all usages: mean 69.0%, standard deviation 19.8, median 71%. With averaging for repeated usages: mean 69.5%, standard deviation 20.7, median 72%). The point biserial ranged from -0.08 – 0.34 (By all usages: mean 0.12, standard deviation 0.08, median 0.11. With averaging for repeated usages: mean 0.11, standard deviation 0.07, median 0.12).

Facility and point biserial results were uncorrelated (-0.04). Similarly, Chi-Square analysis did not identify an association between the binary categorisation of item facility or discrimination (χ ^2^=0.049, 1 degree of freedom, p>0.05), highlighting the independence of these quality markers. Levene’s test for homogeneity was supported for the point biserial for easy or moderate/difficult items (p>0.05).

### Research objective 1 - variation in item statistical performance by item characteristics

The correlation between item characteristics and statistical performance was similar for the 173 unique items and the 293 item usages ( Table 1). Facility was significantly correlated with a vignette being present and required (r=0.21 for items, r=0.22 for usages), diagnostic items (r=0.18) and not being a recall item (r=-0.18 for items, r=-0.21 for usages). For the 293 usages, the length of the stem (r=0.16), clinically-relevant items (r=0.12) and application items (r=0.18) were also significantly positively correlated with facility. However, when the binary classification of item difficulty level and discrimination were analysed ( Table 2), no item characteristics were significantly associated with an item being low difficulty.

**Table 1.** Correlation between item characteristics and item performance. The association between item characteristics and item performance measures (facility and point biserial) was evaluated through linear correlations for five annual summative paediatric medical student assessments. Due to the re-use of some items over the years, results are expressed for all unique items (n=173) and all item usages (n=293). **r** Spearman Rank Order correlation and **p** for a two tailed test * significant at <0.05 ** significant at p<0.01. ‘Usage’ included items being used more than once

Item characteristics	Items (n=173)				Usage (n=293)			
	Average facility		Average point biserial		Facility		Point biserial	
	r	p	r	p	r	p	r	p
Item Variable								
Extended matching question (EMQ)	0.00	0.96	0.22 **	0.00	0.01	0.86	0.18 **	0.00
Longer stem length (number of words)	0.13	0.09	0.00	0.97	0.16 **	0.01	-0.02	0.71
Longer total option length (number of words)	-0.03	0.72	0.16 *	0.04	-0.02	0.77	0.09	0.12
Usage frequency	-0.11	0.15	0.26 **	<0.001	na		na	
Clinical Variable								
Vignette required to answer	0.21 **	0.01	0.07	0.36	0.22 **	<.001	0.07	0.23
One or more writing flaws	-0.08	0.27	0.08	0.29	-0.06	0.35	0.05	0.40
Diagnosis item	0.16 *	0.03	0.18 *	0.02	0.17 **	0.00	0.18 **	0.00
Management item	-0.05	0.50	-0.12	0.12	-0.03	0.62	-0.12 *	0.04
Basic clinical sciences item		0.52	-0.02	0.77	-0.10	0.10	-0.02	0.70
Clinically relevant item	0.14	0.07	0.05	0.50	0.12 *	0.04	0.04	0.49
Bloom’s Taxonomy								
Recall item	-0.18 *	0.02	-0.13	0.09	-0.21 **	<.001	-0.09	0.12
Understanding item	-0.02	0.81	0.03	0.70	-0.04	0.55	-0.01	0.92
Application item	0.14	0.07	0.07	0.38	0.18 **	0.00	0.07	0.23
Content								
Subspecialty item	-0.02	0.80	0.21 **	0.01	-0.04	0.47	0.17 **	0.00
Paediatric surgery item	0.01	0.89	-0.06	0.41	0.02	0.79	-0.02	0.78
General paediatric item	0.01	0.89	-0.16 *	0.04	0.03	0.61	-0.16 **	0.01

**Table 2.** Association of item characteristics with low difficulty and discriminating items (n=173). The item facility and point biserial for all items included in five years of summative paediatric medical student assessments were recorded. Items were grouped according to a binary classification of facility (>80% or ≤80%) and point biserial (<0.15 or ≥0.15). The association between item characteristics for the binary classification of item facility and point biserial was evaluated through Chi-square analysis. **p** is significant at <0.05.

Item characteristic	Items (n=173)			
	Low difficulty item (facility 80% or more)		Good discriminator (point biserial 0.15 or more)	
	Chi- Square	p	Chi- Square	p
Item Type	0.10	0.75	**4.20**	**0.04**
Vignette required to answer	1.90	0.17	1.17	0.28
One or more writing flaws	0.17	0.68	1.01	0.31
Diagnosis item	1.90	0.17	**5.35**	**0.02**
Management item	1.04	0.31	1.71	0.19
Basic clinical item	0.28	0.60	0.18	0.67
Clinically relevant item	0.80	0.37	0.85	0.36
Recall item	1.16	0.28	1.32	0.25
Understanding item	0.05	0.83	0.17	0.68
Application item	0.32	0.57	1.34	0.25
Sub-specialty item	0.12	0.73	1.11	0.29
Surgery item	0.41	0.52	0.13	0.72
Other paediatric item	0.06	0.81	0.58	0.45

Similarly low, but significant, correlations were evident between the point biserial and EMQs (r=0.22 for items, r=0.18 for usages), diagnostic items (r=0.18), subspecialty items (r=0.21 for items, r=0.17 for usages) and not being a general paediatrics item (r=-0.16). A significant but negative correlation was found for the point biserial and management items (r=-0.12) for the 293 item usages. The average point biserial for the 173 items was significantly correlated with the total option length (r=0.16) and usage frequency (r=0.26), reflecting more frequent usage of more discriminating items. EMQ and diagnostic items were again associated with discrimination when analysed using the binary classification of point biserial (p<0.05) ( Table 2).

### Research objective 2 - item characteristics most predictive of statistical performance

Two linear regression analyses were conducted to address this research objective, with facility and point biserial as the dependent variables for the 293 item usages. Only variables identified as having a significant bivariate correlation ( Table 1) were entered into each equation. As shown in Table 3, both regressions were significant at p<0.05, but the amount of variance explained by the 6 item characteristics for facility and the 6 for discrimination was negligible (7% and 8% respectively). The item characteristics used to predict item facility were characterised by more intercorrelation than those used to predict point biserial, as reflected in the Variance Inflation Factor (VIF). As none exceed five, multicollinearity was not sufficient to exclude any variables. No variables in either equation were significant independent predictors, as shown by the t-test results ( Table 3).

**Table 3.** Regression prediction of facility and discrimination (n=293 item usages) by item characteristics. Two linear regression analyses were conducted to determine whether item characteristics were predictive of facility and point biserial for five years of summative paediatric medical student assessments. Only variables identified as having a significant bivariate correlation were entered into each equation. **p** is significant at <0.05. **VIF=** Variance Inflation Factor.

Regression 1	Mean	Standard Deviation	R square	Significance	F
**Facility dependent**	69.03	19.76	0.07	p=0.002	3.48
Predictors	Mean	Standard Deviation	Standardised Beta	t-test p value	VIF
Vignette present and required	0.75	0.44	0.15	NS p>0.05	3.17
Recall item	0.12	0.33	-0.12	NS p>0.05	1.98
Diagnosis item	0.45	0.50	0.10	NS p>0.05	1.26
Clinically relevant item	0.87	0.33	-0.05	NS p>0.05	1.83
Application item	0.69	0.46	-0.03	NS p>0.05	2.66
Stem length (number of words)	51.26	29.17	0.02	NS p>0.05	1.75
Regression 2	Mean	Standard Deviation	R square	Significance	F
**Point biserial dependent**	0.12	0.08	0.08	p<0.001	4.32
Predictors	Mean	Std. Deviation	Standardised Beta	t-test p value	VIF
Diagnosis item	0.45	0.50	0.15	NS p=0.05	1.87
Length of options (number of words)	21.27	10.97	0.13	NS p=0.05	1.30
Other paediatric item	0.31	0.46	-0.12	NS p>0.05	1.65
Subspecialty item	0.42	0.49	0.09	NS p>0.05	1.65
Extended matching question	0.38	0.49	0.05	NS p>0.05	1.41
Management item	0.29	0.45	-0.03	NS p>0.05	1.81

### Research objective 3 - evaluating statistical performance indicators across testing periods

**Figure 2.**
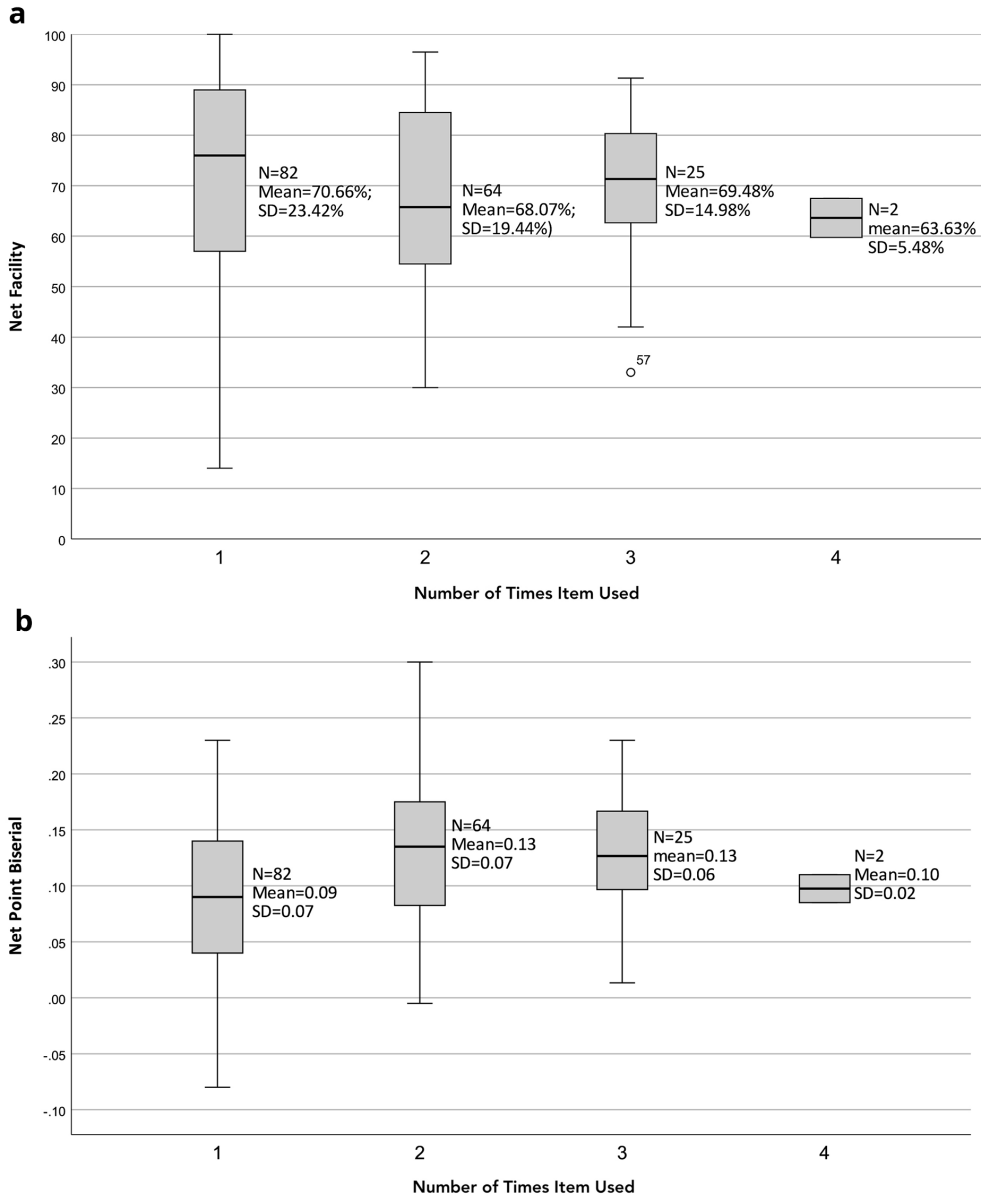
Net item facility ( **a**) and net point biserial ( **b**) with repeated item use. All items used during five consecutive summative paediatric medical student assessments were collated. Some items were re-used during this period and the usage frequency was recorded. The association between usage frequency, net item facility ( **a**) and net point biserial ( **b**) is shown.

Items used twice were more likely to be difficult (mean facility difference -2.6%) and discriminating (point biserial mean difference +0.04) than those used once ( Figure 2). The significant correlation between frequency of use and discrimination (r=0.26, p<0.05) was demonstrated earlier in Table 1. However, there was no association (p>0.05) between usage frequency and whether it was a low difficulty or discriminating item on Chi-squared analyses (χ ^2^=4.57, 3 degrees of freedom and χ ^2^=4.79, 3 degrees of freedom, respectively).

## Discussion

We found two item characteristics were significantly associated with facility for five years of data in our paediatric item bank; use of a vignette required to answer the item and diagnosis items. The presence of a vignette has previously been shown to be associated with item facility ^10^, given that it confers ‘context-richness’ ^7^. The greater facility of diagnostic items is not surprising given that it is a more commonly practiced skill than management in the pre-vocational years. Interestingly, there was also a significant relationship between facility, stem length, clinical relevance and application items for the 293 usages, perhaps because these attributes allow more relevant clinical content to be expressed, although the longest stem was still only 149 words. By contrast, recall items were negatively correlated with item facility, were less likely to have a vignette and were less discriminating. It is worth noting that 3 of the 7 items discarded prior to calculating each students’ score, assessed recall of an epidemiological statistic. This highlights the need to use recall items judiciously. However, the magnitude of the associations was small and these correlations were not reproduced when the data was grouped into ‘low’ or ‘moderate/difficult’ items.

With respect to item discrimination, significant positive associations included EMQ format and items addressing diagnosis or a paediatric subspeciality. These results are consistent with existing studies of the matching question format ^12^. The greater discriminating power of diagnosis items may be due to the focus on assessing skills required for the first post-graduate year. The greater discriminating power of subspeciality paediatric items over general paediatrics items may be an indicator of higher performing students covering more of the content compared with borderline students covering only what was essential to pass. When confining analysis to the 173 items, the point biserial was significantly correlated with total length of options (maximum 132 words) and usage frequency. EMQ and diagnosis items remained significantly associated with point biserial in Chi-squared analyses. As expected, re-used items tended to be at least moderately difficult and discriminating, reflecting re-selection of high-performing items.

Although we identified significant correlations between item characteristics and performance, the size of this effect was small. Less than 10% of the variation in item statistical performance was explained by the item characteristics examined. This requires further study. Although only a small quantum of the item facility and discrimination was explained by modifiable item characteristics, these are some of the easiest item construction variables to modify. Secondly, these data confirm the relative importance of pre-assessment influences, such as teaching content and delivery. Moreover, this result is not unexpected from a highly vetted MCQ examination bank where item construction characteristics should not alter student performance.

This study’s strengths include it being one of few ^10– 12 ^to quantitatively examine the influence of item characteristics over statistical measures of item performance in medical education. Hernandez *et al*. ^12^ addressed the association between item construction, difficulty and discrimination in 125 first year pathology MCQs. Our work focuses on final stage students in paediatrics where image inclusion is less common and vignettes are more frequent and relevant. Our study also included summative items only, a greater number of items (173), over a longer period (5 years) with multiple item coders. Common findings between our studies are that EMQs are more discriminating than SBAs and higher order Bloom’s taxonomic domains (understanding and application items) are not associated with lower item facility and/or discrimination as one might expect. This supports the approach of incorporating items from all strata of the taxonomy. Unlike Hernandez *et al. ^12^*, our study unexpectedly found that recall items were inversely correlated with item facility. One possible explanation for this is that the recall items in their study (96% of all items) were predominantly examining basic science content, whereas a significant portion of the recall items in our study (12% of items) were epidemiological (45%).

Limitations of our study include the dataset being derived from a single specialty rotation at an individual institution. Several item characteristic variables were also removed due to low frequency (item writing flaws, presence of images/tables) but the prevalence and relevance of these characteristics may vary in other settings, limiting generalisability. Although we used a protocol to reduce heterogeneity between item reviewers, we still found variation in item coding. This highlights the subjectivity of this research method, demonstrating the importance of item review by multiple individuals. Future studies could attempt to repeat this study across multiple sites, in a more diverse range of specialities and using items that more frequently include images.

## Conclusions

We found that statistical performance of individual MCQs may be improved through the addition of vignettes, diagnosis items, EMQs, clinically relevant content, longer stems and items requiring knowledge application. In our study, paediatric subspeciality content was associated with greater discrimination than general paediatrics content, with recall and management items being negatively associated with facility and discrimination respectively. Item preparation should be informed by these findings. Nonetheless, the magnitude of these associations was small, highlighting the importance of pre-examination pedagogical influences.

## Data Availability

Figshare: Assessment metric data from items used for a paediatric end of block MCQ exam across 5 years: Statistical performance paediatric assessment data_README.txt. https://doi.org/10.6084/m9.figshare.24037767 ^16^. Figshare: Assessment metric data and statistical performance of all unique items used for a paediatric end of block MCQ exam across 5 years (n=173): CAH MCQ N293 Item Usage.csv. https://doi.org/10.6084/m9.figshare.24037662 ^17^. Figshare: Assessment metric data and statistical performance of all items used (all ‘usages’) for a paediatric end of block MCQ exam across 5 years (n=293): CAH MCQ N173 Unique Items.csv. https://doi.org/10.6084/m9.figshare.24037659 ^18^. Data are available under the terms of the Creative Commons Zero "No rights reserved" data waiver (CC0 1.0 Public domain dedication).

## References

[1] Pugh D De Champlain A Touchie C : Plus ça change, plus c'est pareil: Making a continued case for the use of MCQs in medical education. *Med Teach.* 2019;41(5):569–577. 10.1080/0142159X.2018.1505035 30299196

[2] Javaeed A : Assessment of Higher Ordered Thinking in Medical Education: Multiple Choice Questions and Modified Essay Questions [version 1]. *MedEdPublish.* 2018;7: 128. Reference Source 10.15694/mep.2018.0000128.1 PMC10699377 38074575

[3] Norcini J Anderson MB Bollela V : 2018 Consensus framework for good assessment. *Med Teach.* 2018;40(11):1102–1109. 10.1080/0142159X.2018.1500016 30299187

[4] Campbell DE : How to write good multiple-choice questions. *J Paediatr Child Health.* 2011;47(6):322–325. 10.1111/j.1440-1754.2011.02115.x 21615597

[5] Paniagua MA Swygert KA : Constructing Written Test Questions For the Basic and Clinical Sciences.In: 5 ed. Philadelphia: National Board of Medical Examiners;2016; Accessed 26/02/2022. Reference Source

[6] Case S Swanson D : Constructing Written Test Questions For the Basic and Clinical Sciences. National Board of Examiners.2002. Reference Source

[7] Epstein RM : Assessment in Medical Education. *N Engl J Med.* 2007;356(4):387–396. 10.1056/NEJMra054784 17251535

[8] Jozefowicz RF Koeppen BM Case S : The quality of in-house medical school examinations. *Acad Med.* 2002;77(2):156–161. 10.1097/00001888-200202000-00016 11841981

[9] Bibler Zaidi NL Grob KL Monrad SU : Item Quality Improvement: What Determines a Good Question? Guidelines for Interpreting Item Analysis Reports. *Medical Science Educator.* 2018;28(1):13–17. 10.1007/s40670-017-0506-1

[10] Ikah DS Finn GM Swamy M : Clinical vignettes improve performance in anatomy practical assessment. *Anat Sci Educ.* 2015;8(3):221–229. 10.1002/ase.1471 24953193

[11] Holland J O’Sullivan R Arnett R : Is a picture worth a thousand words: an analysis of the difficulty and discrimination parameters of illustrated vs. text-alone vignettes in histology multiple choice questions. *BMC Med Educ.* 2015;15(1):184. 10.1186/s12909-015-0452-9 26502882 PMC4623296

[12] Hernandez T Magid MS Polydorides AD : Assessment Question Characteristics Predict Medical Student Performance in General Pathology. *Arch Pathol Lab Med.* 2021;145(10):1280–1288. 10.5858/arpa.2020-0624-OA 33450752

[13] Cook AK Lidbury JA Creevy KE : Multiple-Choice Questions in Small Animal Medicine: An Analysis of Cognitive Level and Structural Reliability, and the Impact of these Characteristics on Student Performance. *J Vet Med Educ.* 2020;47(4):497–505. 10.3138/jvme.0918-116r 32163022

[14] Tukey JW : Exploratory data analysis.Reading, Mass.: Addison-Wesley Pub. Co.;1977. Reference Source

[15] Feldman K Chawla N : Does Medical School Training Relate to Practice? Evidence from Big Data. *Big Data.* 2015;3(2):103–113. 10.1089/big.2014.0060 26487985 PMC4605456

[16] Clark T : Statistical performance paediatric assessment data_README.txt. figshare. Online resource,2023. 10.6084/m9.figshare.24037767.v1

[17] Clark T Bosi I O'Mara D : CAH MCQ N173 Unique Items.csv. figshare. [Dataset],2023. 10.6084/m9.figshare.24037659.v1

[18] Clark T : CAH MCQ N293 Item Usage.csv. figshare. [Dataset],2023. 10.6084/m9.figshare.24037662.v1

