# Supplementary material for: Associations between item characteristics and statistical performance for paediatric medical student multiple choice assessments
Source: MedEdPublish (2016). 2023 Nov 8;13:270. [Version 1] doi: 10.12688/mep.19764.1 (PMC11253842; doi:10.12688/mep.19764.1)
Supplement: Peer review history

# Reviewer report: Reviewer response for version 1

**DOI:** 10.21956/mep.21178.r36797

**Published:** 2024-07-12

**Authors:** Jane Holland

**Affiliations:** 1 University of Medicine and Health Sciences, Dublin, Ireland

Thank you for an interesting paper. It covers a lot of ground, which can make such a paper challenging to decide what background context and references to include, without being excessive. Nonetheless, MedEdPublish is a scholarly journal, and as such, published papers should thoroughly reference present and discuss the existing evidence-base in relation to the original research findings, both to place the findings in context, and so that interested readers may read further about various factors or facets of the paper as they wish.

**Introduction**

 There have been a number of papers published about MCQ writing over the past two decades, and institutions such as NBME have extensive internal analyses also, so I disagree that *'existing MCQ guidelines are largely derived from consensus rather than quantitative evidence*' (Swanson, Case et al. 2001, Wood and Cole 2001, Haladyna, Downing et al. 2002, NBME 2021).

 There are a number of papers exploring the influence of item flaws, which could be referenced and discussed in comparison to these findings, including:

- Haladnya, Costello, Casu, Pais, Rodrí­guez-Díez, Rush, Tarrant, Ali et al (Haladyna and Downing 2004, Tarrant, Knierim et al. 2006, Tarrant and Ware 2008, Ali and Ruit 2015, Pais, Silva et al. 2016, Rodrí­guez-Díez, Alegre et al. 2016, Rush, Rankin et al. 2016, Casu and García-Garcíab 2018, Costello, Holland et al. 2018, NBME 2021)
- THE NBME have updated their Guidelines since 2002, which are downloadable online as far as I know, the current reference is *NBME (2021). Constructing written test questions for the basic and clinical sciences, National Board of Medical Examiners Philadelphia, PA.*

Additionally, there have been papers published comparing 5 option SBAs to EMQs, if wishing to make note of this, the ones I would recommend reading & referring to is that by Coderre and/or Swanson (Coderre, Harasym et al. 2004, Swanson, Holtzman et al. 2006). Of course, SBAs can use different numbers of options (my institution has moved from five to three for some programmes), so long as the cut score is adjusted, or the assessment design modified to account for moving from a 20% to 33% guess rate (Swanson, Holtzman et al. 2005, Tarrant and Ware 2010, Schneid, Armour et al. 2014).

 Sylvain Coderre has also written on testing Clinical Reasoning in MCQs, as have Heist, Beullens and others, which could be included.(Beullens, Van Damme et al. 2002, Coderre, Mandin et al. 2003, Beullens, Struyf et al. 2005, Heist, Gonzalo et al. 2014).

 A number of authors other than Holland et al have explored the influence of images in MCQs, which I think could also be referenced: (Vorstenbosch, Klaassen et al. 2013, Sagoo, Vorstenbosch et al. 2021)

 Notebaert makes reference both to his findings on images in MCQs, and the manner in which he used to compare items from different cohorts - no need to duplicate this, but you should mention it as an alternative method of analysis, and give some brief justification as to why you used the methodology by Hernandez instead (Notebaert 2016).

**Methods / results:**

1. The various statistical analyses are quite dense to review, even with experience in psychometrics, and I would advise giving some references on interpretation of psychometrics, perhaps again within the introduction, but certainly within the discussion as appropriate (De Champlain 2010, Tavakol and Dennick 2011, Bibler Zaidi, Grob et al. 2017).
2. The RPB in this study seem low, and should be commented upon as a valuable piece of information for quality improvement. It is generally advised that RPB be > 0.2 for assessments (Beichner 1994, Engelhardt 2009, Bibler Zaidi, Grob et al. 2017).
3. I suggest adjusting the sentence at the end of Page 4, column 2, paragraph 4 to 'EMQ and diagnostic items were again associated with *higher* discrimination when analysed'
4. For Research Objective 3, were the Facility and Discrimination of these items known when they were chosen for reuse? My assumption is that their performance statistics were likely the very reason for their reuse?

Have any limitations of the research been acknowledged?

Yes

Is the work clearly and accurately presented and does it cite the current literature?

Partly

If applicable, is the statistical analysis and its interpretation appropriate?

I cannot comment. A qualified statistician is required.

Are all the source data underlying the results available to ensure full reproducibility?

Yes

Is the study design appropriate and is the work technically sound?

Yes

Are the conclusions drawn adequately supported by the results?

Yes

Are sufficient details of methods and analysis provided to allow replication by others?

Yes

Reviewer Expertise:

Anatomy & Assessment. While I'm very familiar with the practical use of item statistics (and deliver some training on them) I'm neither a statistician nor a psychometrician, so for original research aspects such as the linear analyses and multicollinearity are beyond my expertise and should have further specialist peer review.

I confirm that I have read this submission and believe that I have an appropriate level of expertise to confirm that it is of an acceptable scientific standard.

## References

[1] : The Impact of item flaws, testing at low cognitive level, and low distractor functioning on multiple-choice question quality. *Perspect Med Educ* .2015;4(5) : 10.1007/s40037-015-0212-x 244-251 10.1007/s40037-015-0212-x 26350082 PMC4602009

[2] : Testing student interpretation of kinematics graphs. *American Journal of Physics* .1994;62(8) : 10.1119/1.17449 750-762 10.1119/1.17449

[3] : Do extended matching multiple-choice questions measure clinical reasoning?. *Med Educ* .2005;39(4) : 10.1111/j.1365-2929.2005.02089.x 410-7 10.1111/j.1365-2929.2005.02089.x 15813764

[4] : Item Quality Improvement: What Determines a Good Question? Guidelines for Interpreting Item Analysis Reports. *Medical Science Educator* .2018;28(1) : 10.1007/s40670-017-0506-1 13-17 10.1007/s40670-017-0506-1

[5] : Differential Length and Overlap with the Stem in Multiple-Choice Item Options: A Pilot Experiment. *Psicología Educativa* .2018;25(1) : 10.5093/psed2018a20 43-48 10.5093/psed2018a20

[6] : The impact of two multiple-choice question formats on the problem-solving strategies used by novices and experts. *BMC Med Educ* .2004;4: 10.1186/1472-6920-4-23 23 10.1186/1472-6920-4-23 15530166 PMC533882

[7] : Diagnostic reasoning strategies and diagnostic success. *Med Educ* .2003;37(8) : 10.1046/j.1365-2923.2003.01577.x 695-703 10.1046/j.1365-2923.2003.01577.x 12895249

[8] : The future of online testing and assessment: question quality in MOOCs. *International Journal of Educational Technology in Higher Education* .2018;15(1) : 10.1186/s41239-018-0124-z 10.1186/s41239-018-0124-z

[9] : A primer on classical test theory and item response theory for assessments in medical education. *Med Educ* .2010;44(1) : 10.1111/j.1365-2923.2009.03425.x 109-17 10.1111/j.1365-2923.2009.03425.x 20078762

[10] : Construct‐Irrelevant Variance in High‐Stakes Testing. *Educational Measurement: Issues and Practice* .2004;23(1) : 10.1111/j.1745-3992.2004.tb00149.x 17-27 10.1111/j.1745-3992.2004.tb00149.x

[11] : Exploring Clinical Reasoning Strategies and Test-Taking Behaviors During Clinical Vignette Style Multiple-Choice Examinations: A Mixed Methods Study. *J Grad Med Educ* .2014;6(4) : 10.4300/JGME-D-14-00176.1 709-14 10.4300/JGME-D-14-00176.1 26140123 PMC4477567

[12] : The effect of images on item statistics in multiple choice anatomy examinations. *Anat Sci Educ* .2017;10(1) : 10.1002/ase.1637 68-78 10.1002/ase.1637 27472765

[13] : Do item-writing flaws reduce examinations psychometric quality?. *BMC Res Notes* .2016;9(1) : 10.1186/s13104-016-2202-4 399 10.1186/s13104-016-2202-4 27516160 PMC4982015

[14] : Technical flaws in multiple-choice questions in the access exam to medical specialties ("examen MIR") in Spain (2009-2013). *BMC Med Educ* .2016;16: 10.1186/s12909-016-0559-7 47 10.1186/s12909-016-0559-7 26846526 PMC4741005

[15] : The impact of item-writing flaws and item complexity on examination item difficulty and discrimination value. *BMC Med Educ* .2016;16(1) : 10.1186/s12909-016-0773-3 250 10.1186/s12909-016-0773-3 27681933 PMC5041405

[16] : Online Assessment of Applied Anatomy Knowledge: The Effect of Images on Medical Students' Performance. *Anat Sci Educ* .2021;14(3) : 10.1002/ase.1965 342-351 10.1002/ase.1965 32289198

[17] : Reducing the number of options on multiple-choice questions: response time, psychometrics and standard setting. *Med Educ* .2014;48(10) : 10.1111/medu.12525 1020-7 10.1111/medu.12525 25200022

[18] : Relationships among item characteristics, examine characteristics, and response times on USMLE Step 1. *Acad Med* .2001;76(10 Suppl) : 10.1097/00001888-200110001-00038 S114-6 10.1097/00001888-200110001-00038 11597892

[19] : Psychometric characteristics and response times for content-parallel extended-matching and one-best-answer items in relation to number of options. *Acad Med* .2006;81(10 Suppl) : 10.1097/01.ACM.0000236518.87708.9d S52-5 10.1097/01.ACM.0000236518.87708.9d 17001136

[20] : Psychometric characteristics and response times for one-best-answer questions in relation to number and source of options. *Acad Med* .2005;80(10 Suppl) : 10.1097/00001888-200510001-00025 S93-6 10.1097/00001888-200510001-00025 16199468

[21] : The frequency of item writing flaws in multiple-choice questions used in high stakes nursing assessments. *Nurse Educ Today* .2006;26(8) : 10.1016/j.nedt.2006.07.006 662-71 10.1016/j.nedt.2006.07.006 17014932

[22] : Impact of item-writing flaws in multiple-choice questions on student achievement in high-stakes nursing assessments. *Med Educ* .2008;42(2) : 10.1111/j.1365-2923.2007.02957.x 198-206 10.1111/j.1365-2923.2007.02957.x 18230093

[23] : A comparison of the psychometric properties of three- and four-option multiple-choice questions in nursing assessments. *Nurse Educ Today* .2010;30(6) : 10.1016/j.nedt.2009.11.002 539-43 10.1016/j.nedt.2009.11.002 20053488

[24] : Do images influence assessment in anatomy? Exploring the effect of images on item difficulty and item discrimination. *Anat Sci Educ* .2013;6(1) : 10.1002/ase.1290 29-41 10.1002/ase.1290 22674609

[25] : An Introduction to Classical Test Theory as Applied to Conceptual Multiple-choice Tests. *https://www.per-central.org/items/detail.cfm?ID* .2009;

[26] : A review of multiple-choice item-writing guidelines for classroom assessment. *https://psycnet.apa.org/record/2002-15954-005* .2002;

[27] : Constructing written test questions for the basic and clinical sciences. *https://health.uconn.edu/faculty-development/wp-content/uploads/sites/69/2017/06/constructing_written_test_questions.pdf* .

[28] Developing MultipleChoice Questions for the Royal College Certification Examinations. *https://cdn.wildapricot.com/228591/resources/Documents/GuidelinesforDevelopmentMCQRoyalCollege.pdf?version* .

[29] : Constructing Written Test Questions For the Basic and Clinical Sciences. *https://www.researchgate.net/publication/242759434_Constructing_Written_Test_Questions_For_the_Basic_and_Clinical_Sciences* .2002;

[30] : Constructing Written Test Questions For the Basic and Clinical Sciences. *https://case.edu/medicine/caml/sites/default/files/2018-04/Constructing-test-questions-NBME.pdf* .1998;

# Reviewer report: Reviewer response for version 1

**DOI:** 10.21956/mep.21178.r36799

**Published:** 2024-05-09

**Authors:** Danny Mathysen

**Affiliations:** 1 Antwerp University Hospital, Antwerp, Belgium

The authors have written very interesting research results. I understand that the number of items under investigation presenting one or more item flaws were rather limited. It would be interesting to see in a larger dataset what would be the results of a similar study in items with item flaws compared to items without items flaws.

 I have a couple of questions for the authors to be clarified:

 1. Why have some items been re-used knowing they had been excluded before?

 2. Why is there a difference in the cut-off of the point-biserial correlation coefficient when it comes to post administration review (negative point biserial) and classification of items according to point biserial (cut-off of 0.15)?

 3. Regarding the identification of item flaws, it is not clear whether they have been indicated on an individual basis, or whether these individual indications had later on been agreed upon by the three coders.

 4. Regarding figure 1: were there SBA's included with more than 5 item options?

 5. Regarding figure 1: are stem length and length of options counted in words?

 6. Regarding figure 1: clinical relevance coding was described in the text as low-moderate-high, while in the graph "low" is not present

 7. Regarding figure 1: item flaws have been concluded in the text as a binary option, while in the figure it is presented otherwise

Have any limitations of the research been acknowledged?

Yes

Is the work clearly and accurately presented and does it cite the current literature?

Yes

If applicable, is the statistical analysis and its interpretation appropriate?

Yes

Are all the source data underlying the results available to ensure full reproducibility?

Yes

Is the study design appropriate and is the work technically sound?

Yes

Are the conclusions drawn adequately supported by the results?

Yes

Are sufficient details of methods and analysis provided to allow replication by others?

Yes

Reviewer Expertise:

psychometric analysis

I confirm that I have read this submission and believe that I have an appropriate level of expertise to confirm that it is of an acceptable scientific standard.
